# PHL7/441: Fixing a Broken Line between the Perceived "Anarchy" of the Web and a Process-Comfortable Pharmaceutical Company

**DOI:** 10.2196/jmir.1.suppl1.e88

**Published:** 1999-09-19

**Authors:** L Vercellesi

**Affiliations:** ^1^Zeneca, BasiclioItaly

**Keywords:** Drug Industry, Pharmaceutical Information, Standard Operating Procedures, Guidelines, Webmaster

## Abstract

**Introduction:**

In 1998 a pharmaceutical company published its Web site to provide:

- an institutional presence
- multifunctional information to primary customers and general public
- a new way of access to the company
- a link to existing company-sponsored sites
- a platform for future projects

Since the publication, some significant integration have been added; in particular one is a primary interactive service, addressed to a selected audience. The need has been felt to foster new projects and establish the idea of routinely considering the site as a potential tool in the marketing mix, to provide advanced services to customers.

**Methods:**

Re-assessment of the site towards objectives. Assessment of its perception with company potential suppliers.

**Results:**

The issue "web use" was discussed in various management meetings; the trend of use of Internet among the primary customers was known; major concerns expressed were about staffing and return of investment for activities run in the Web. These perceptions are being addressed by making the company more comfortable by:

- Running the site through a detailed process and clear procedures, defining
  1. A new process of maintenance of the site, involving representatives of all the functions.
  2. Procedures and guidelines.
  3. A master file of approved answers and company contacts.
  4. Categories of activities (information, promotion, education, information to investors, general services, target-specific services).
  5. Measures for all the activities run in the Web site
- Specifically for the Web site a concise periodical report is being assessed, covering
  1. Statistics about hits and mails, compared to the corporate data.
  Indication of new items published.
  Description by the "supplier" of new or ongoing innovative projects, to transfer best practice.
  Basic figures on the Italian trend in internet use and specifically in the pharmaceutical and medical fields.
  Comments to a few competitor sites.
  Examples of potential uses deriving from other Web sites.

**Discussion:**

The comparatively low use of Internet in Italy has affected the systematic professional exploitation of the company site. The definition of "anarchic" commonly linked to the Web by local media has lead to the attempt to "master" and "normalize" the site with a stricter approach than usual: most procedures and guidelines have been designed from scratch as not available for similar activities traditionally run. A short set of information has been requested for inclusion in the report: its wide coverage will help to receive a flavour of the global parallel new world developing in the net. Hopefully this approach will help to create a comfortable attitude towards the medium in the whole organisation and to acquire a working experience with the net.

